# Decentering, Acceptance, and Non-Attachment: Challenging the Question “Is It Me?”

**DOI:** 10.3389/fpsyt.2021.659835

**Published:** 2021-11-18

**Authors:** Joaquim Soler, Jesus Montero-Marin, Elisabet Domínguez-Clavé, Sara González, Juan Carlos Pascual, Ausiàs Cebolla, Marcelo Demarzo, Bhikkhu Analayo, Javier García-Campayo

**Affiliations:** ^1^Servei de Psiquiatria, Hospital de la Santa Creu i Sant Pau, Barcelona, Spain; ^2^Department of Psychiatry and Forensic Medicine, Universitat Autònoma de Barcelona (UAB), Barcelona, Spain; ^3^Centro de Investigación Biomédica en Red de Salud Mental, CIBERSAM, Madrid, Spain; ^4^Department of Psychiatry, Warneford Hospital, University of Oxford, Oxford, United Kingdom; ^5^Department of Pharmacology and Therapeutics, Universitat Autònoma de Barcelona (UAB), Barcelona, Spain; ^6^Departamento de Personalidad, Evaluación y Tratamientos Psicológicos, University of Valencia UV, València, Spain; ^7^CIBER Physiopathology of Obesity and Nutrition (CIBEROBN), Madrid, Spain; ^8^Mente Aberta – Brazilian Center for Mindfulness and Health Promotion, Universidade Federal de São Paulo (UNIFESP), São Paulo, Brazil; ^9^Barre Center of Buddhist Studies (BCBS), Barre, MA, United States; ^10^Numata Center for Buddhist Studies, University of Hamburg, Hamburg, Germany; ^11^Red de Investigación de Actividades Preventivas y Promoción de la Salud (RedIAPP), Zaragoza, Spain; ^12^Miguel Servet Hospital, University of Zaragoza, Zaragoza, Spain

**Keywords:** mindfulness, acceptance, decentering, non-attachment, depression, resilience

## Abstract

Among mindfulness measures the three constructs acceptance, decentering, and non-attachment are psychometrically closely related, despite their apparent semantic differences. These three facets present robust psychometric features and can be considered core themes in most “third wave” clinical models. The aim of the present study was to explore the apparently different content domains (acceptance, decentering, and non-attachment) by administering various psychometric scales in a large sample of 608 volunteers. Resilience and depression were also assessed. Exploratory and confirmatory factor analyses performed in two randomly selected subsamples showed a bifactor approximation. The explained common variance suggested a unidimensional nature for the general factor, with good psychometric properties, which we named “Delusion of Me” (DoM). This construct is also strongly correlated with resilience and depression, and appears to be a solid latent general construct closely related to the concept of “ego.” DoM emerges as a potentially transdiagnostic construct with influence on well-being and clinical indexes such as resilience and depression. Further studies should analyze the potential utility of this new construct at a therapeutic level.

## Introduction

In recent years, mindfulness has become an important health-related concept, and the absence of mindfulness has been consistently associated with clinical symptoms, both medical and psychological (1, 2). By contrast, the presence of mindfulness has been positively correlated with quality of life and well-being (3–6). Traditionally, mindfulness is considered to contain two main components: (1) self-regulation of attention and (2) an attitude of acceptance (7). Currently available measures of mindfulness are highly heterogeneous assessing as few as a single characteristic of mindfulness (8) to up to nine distinctive features (9). Given this heterogeneity, together with other controversial aspects such as diverse theoretical backgrounds, differences in the definitions used in these scales, the trait or state debate, and the question of to what extent mindfulness is trainable (10–13), it is not surprising that relationship between the various mindfulness scales is generally poor, particularly in the case of dispositional mindfulness and non-experienced populations (14). Importantly, this lack of consistency between these instruments does not apply to all of the sub-facets and by-products of mindfulness practice. In fact, certain aspects such as acceptance, decentering, and non-attachment are all highly correlated.

Several instruments are available to assess measures for each of these three domains. In the most commonly used mindfulness questionnaire—the “Five Facets Mindfulness Questionnaire” [FFMQ, (15)]—acceptance is best represented by accepting without judgment, which refers to taking a non-evaluative stance toward the feelings and thoughts. Nevertheless, the developer of the FFMQ originally suggested that non-reacting together with non-judging of inner experience both represent acceptance (16). Several studies have shown a significant overlap between the non-reacting and the decentering constructs, which consistently correlate with each other, even more so than with the non-judging facet (10, 17, 18). Likewise, the non-judging subscale strongly correlates with other measures of acceptance, such as experiential avoidance in the “Acceptance and Action Questionnaire-II” (AAQ-II) and the acceptance subscale in the “Philadelphia Mindfulness Scale” (PHLMS) (1, 19, 20).

Decentering, also referred to as defusion, reification, or meta-awareness in the literature, is commonly assessed with the Experiences Questionnaire [EQ, (21)]. However, decentering is also evaluated in other instruments, including the Toronto Mindfulness Scale [TMS, (22)], the Southampton Mindfulness Questionnaire [SMQ, (23)], and the Comprehensive Inventory of Mindfulness Experience [CHIME, (20)]. Decentering has been defined as “the ability to observe one's thoughts and feelings in a detached manner, as temporary events in the mind, as neither necessarily true nor reflections of the self” (24). Studies have shown that a low level of decentering can, by itself, predict an earlier relapse in individuals who have recently recovered from major depression (25). Some reports also suggest that decentering may be impaired in individuals with borderline personality disorder, eating behavior disorder, or cocaine dependence (26). Decentering is closely related to measures of acceptance such as experiential avoidance and non-acceptance, both included in the Difficulties in Emotion Regulation Scale (DERS), and with non-judging from the FFMQ (27, 28).

A final but equally important element is non-attachment, which has its origins in Buddhism. Non-attachment can be defined as the relative absence of fixation on ideas, images, or sensory objects and as an absence of internal pressure to obtain, hold, avoid, or change circumstances or experiences (29). This construct has been positively associated with meditation practice (total weekly hours and frequency of practice), suggesting that regular mindfulness practice may promote non-attachment (27). The Non-Attachment Scale [NAS, (29)] is an accurate representation of non-attachment, and as with decentering, it is closely correlated with acceptance (29), as assessed with the Acceptance and Action Questionnaire-II [AAQ-II, (30)] and the FFMQ non-judging facet (27, 31). Feliu-Soler et al. (27) found a close association between non-attachment and decentering, and Allen (32) suggested that decentering appears to be a quality embedded in non-attachment. Furthermore, non-attachment to the self—which refers to a decrease in self-referential processing or Experiential Selfless Processing (33), that is, processing the present moment subjective experience without self-referentiality—has been identified as a core mechanism in mindfulness training (34–36).

It could be argued that acceptance, decentering, and non-attachment are all intimately connected, not only psychometrically but also theoretically. “If one grasps onto the true existence object [by fusion], this gives rise to attachment” (37). The unaware identification with the content of perception and thought is one contemporary conceptualization of the term “ego” (38). This leads to the confusion of our mental representations with ourselves. As the self-verification theory suggests, people strongly identify with those representations and foster, protect, and defend those self-images (39).

When we do not accept something, we are judging it to be good or bad, and this judgment is fused with a particular desired outcome that seeks our “own” benefit. Some core themes in mindfulness-based programs (MBPs) partially represent this overlapping nature, which can be seen in the concepts of “aversion” and “craving” in mindfulness-based cognitive therapy (MBCT), the idea of “letting go” in both mindfulness-based stress reduction (MBSR) and MBCT, and in radical acceptance principles and techniques such as “willingness vs. willfulness” in dialectical behavior therapy (DBT). In all cases, one relinquishes involvement and frees oneself from the need for things to be different (40–42). These attitudinal components, which are closely related to acceptance, could be more stable and reliable characteristics than the attentional component of mindfulness, which appear to be mood dependent (2). Given the theoretical and psychometric correlation among acceptance, decentering, and non-attachment, we hypothesized that these concepts may, in fact, be different semantic expressions, or the building blocks, of the same latent construct. Therefore, we believe that this new construct could be central to several views of “ego” where fusion with mental content is a key feature, such as the self-as-object (38), the analytical self (43), the narrative self (44, 45), or the self-as-content (46). If this construct is reliable, it would allow us to better understand a form of self that, when becomes overidentified, can be the cause of suffering (38). The idea of a unified self with a sense of agency relies on a coherent set of beliefs, intentions, and behaviors, and is also represented in several contemplative traditions (47) that have been taken as a source of inspiration for some evidence based contemporaneous psychotherapies (40–42). Those traditions advocate for the idea of non-self and actions performed, or motivated by lack of desire (48–50). In all three cases attachment, fusion, and non-acceptance are part of the equation.

To explore this hypothesis, we sought to analyze the possible unidimensionality of a general factor underlying acceptance, decentering, and non-attachment using the non-judging facet included in the FFMQ, EQ, and NAS scales. Second, we also hypothesized that this new general construct, which may subsume these three facets, would be positively correlated with well-being and negatively correlated with psychopathology. Therefore, the aim of the present study was to define this construct and then to perform a preliminary test of its nomological validity compared with an important positive psychology index, resilience, and to depressive symptoms.

## Materials and Methods

A Cross-sectional study was developed with a convenience sample of Spanish general population participants that were included if: (1) aged >18 years, (2) able to understand Spanish, and (3) signed informed consent.

### Participants and Procedure

Participants were recruited through several Spanish websites. An internet-based commercial system specifically designed to recruit survey samples was used to recruit our sample (www.surveymonkey.com; Portland, OR, USA). A link containing the assessment protocol was posted on mindfulness and meditation focused portals and several mindfulness associations, *sanghas*, and Zen monasteries. The non-meditative convenience samples were enrolled accessing to the link placed in scientific and psychology research sites and non-professional social networks (e.g., Facebook). The study protocol was approved by the corresponding regional health authority, the Aragon Ethics Committee (CEICA), Spain (registry: PI12/00083) and all participants signed a consent form indicating their willingness to participate. Prospective participants were invited to participate voluntarily in the study and were told that there would be no monetary compensation. Participants were informed about the purpose of the study and informed that all responses would be treated confidentially. Scales were filled online, with the possibility of using mobile phones or computers, and time taken was around 20 min. A total of 850 subjects voluntarily agreed to participate; of these, 608 (72%) completed all of the requirement instruments and questionnaires. Two randomly selected halves of this sample, with a size of n1 = 304 and n2 = 304, supposing a null hypothesis that RMSEA would be equal to or <0.04 if the true value was 0.06 (close fit) and an alpha equal to 0.05 level, under the more conservative model tested (i.e., only one factor that covers all the items), produces a power coefficient of 0.99 for the exploratory and confirmatory factor analyses. The socio-demographic characteristics of the sample and the randomly-selected subsamples are described in Table 1.

**Table 1:** General socio-demographic variables of the participants according to subsample.

**Variables/samples**		**Total sample**		**Subsample 1**		**Subsample 2**		***p***
		**(*n* = 608)**		**(*n* = 304)**		**(*n* = 304)**		
	**Range**	**Mean**	**SD**	**Mean**	**SD**	**Mean**	**SD**	
Age, years*	17–75	41.32	11.13	40.91	11.52	41.72	10.72	0.379
	**Category**	**Freq**.	**%**	**Freq**.	**%**	**Freq**.	**%**	
Gender**	Female	374	63.2	191	64.5	183	61.8	0.495
	Male	218	36.8	105	35.5	113	38.2	
Education**	Primary	11	1.9	6	2.0	5	1.7	9.982
	Secondary	117	19.8	57	19.3	60	20.3	
	Diploma	86	14.5	45	15.2	41	13.9	
	Degree	300	50.7	150	50.7	150	50.7	
	Ph.D.	78	13.2	38	12.8	40	13.5	
Meditation**	Yes	335	56.6	160	54.1	175	59.1	0.214
	No	257	43.4	136	45.9	121	40.9	

*SD, standard deviation; Freq, frequencies; %, percentages.*

*18 missing values (9 in subsamples 1 and 9 in subsample 2).

**16 missing values (8 in subsamples 1 and 8 in subsample 2).

### Measures

First, we collected information on age, gender (male, female), education (primary, secondary, Associate's degree, Bachelor's degree, Ph.D.), and previous experience with meditation (yes or no).

The Spanish version of the FFMQ (16, 19) is a tool designed to evaluate mindfulness. For the purposes of the present study, we used only the “non-judging of inner experience” dimension of the FFMQ. This dimension comprises eight items (e.g., “I make judgments about whether my thoughts are good or bad”) that are designed to assess an individual's ability to take a non-evaluative stance toward experience. In view of the existing debate surrounding the most appropriate measure of acceptance—traditionally associated with non-judging and non-reactivity FFMQ subscales (18, 20, 51)—we elected to use only the first subscale as this is more closely associated with other acceptance measures than the FFMQ non-reactivity subscale, both in clinical and non-clinical samples (1). Participants were asked to rate each sentence on a five-point scale ranging from 1 (“never or very rarely true”) to 5 (“very often or always true”), with higher scores indicating higher levels of non-judging of inner experience. The Spanish language version of the FFMQ scale and subscales has shown appropriate psychometric properties (19). The composite reliability of non-judging inner experience in the present study sample was ω = 0.93.

The EQ (21, 26) is used to assess the ability of an individual to observe their own thoughts and emotions as temporary objects of the mind, captured under the concept of “decentering.” The EQ contains 11 items designed to measure a metacognitive ability known as “decentering,” defined as the capacity to observe one's thoughts and emotions in a detached manner, considering them transient events of the mind (e.g., “I can separate myself from my thoughts and feelings”). Two original items from the EQ (“I can treat myself kindly” and “I can slow my thinking at times of stress”) were not included in the present analysis because the divergent meaning of these items appears to be more closely related to the self-compassion construct (52). Thus, in the present study, we used a nine-item version of the EQ scale. All items on the EQ are scored on a five-point scale, ranging from 1 (“never”) to 5 (“all the time”), with higher scores indicating a greater decentering capacity. The Spanish language version of the EQ has shown adequate psychometric properties (26). The composite reliability of the final nine decentering items in the present study was ω = 0.87.

The NAS-7 (31) is a 7-item measure extracted from the original 30-item NAS. The original NAS-30 (29) was empirically derived from a pool of items obtained from Buddhist texts about non-attachment. The NAS-7 is a unidimensional short form that measures the absence of fixation on ideas, images, or sensory objects, as well as the absence of internal pressure to obtain, hold, avoid, or change circumstances or experiences. Items (e.g., “I can let go of regrets and feelings of dissatisfaction about the past”) are scored on a six-point scale ranging from 1 (“strongly disagree”) to 6 (“strongly agree”), with higher scores indicating greater levels of non-attachment. NAS-7 has shown good internal consistency for both meditators and non-meditators (27). The composite reliability of non-attachment in the present study was ω = 0.85.

The Connor-Davidson Resilience Scale-10 [CD-RISC-10 (53, 54)] is a self-administered 10-item measure designed to assess a broad construct of resilience (e.g., “I can deal with whatever comes”), a protective factor against mental problems that is positively associated with adaptive coping. Items from the CD-RISC-10 range from 0 (“not true at all”) to 4 (“true nearly all the time”). There is a total scale score, with higher scores indicating greater resilience. The original study on the development of the CD-RISC in the general population and inpatients provided support for the internal consistency, test–retest reliability, and validity of this scale (53). The composite reliability of the CD-RISC-10 scale in the present study was good, with a value of ω = 0.91.

The Depression Anxiety Stress Scale, short form [DASS-21 (55, 56)], is a self-administered, 21-item instrument with three subscales, which assesses depressive symptomatology, anxiety, and stress. In the present study, we used only the depression subscale (e.g., “life is meaningless”). The items in that subscale assess the severity/frequency of symptoms over the previous week on a four-point scale ranging from 0 (“nothing applicable to me”) to 3 (“very applicable to me”), with higher scores indicating greater depressive symptoms. The composite reliability of the depression subscale in the present study was adequate, with a value of ω = 0.90.

The selection of 24 items, coming from FMMQ, EQ and NAS-7, which are the components of the new construct studied, are specified in the Supplementary Material.

### Data Analyses

Sociodemographic data were described as means with standard deviation (SD) and frequencies (percentages) according to the statistical distribution of each variable. Possible differences between two randomly selected subsamples were tested using *t*-tests for independent groups and χ^2^ (or Fisher's exact test when necessary) tests depending on the shape of the score distributions of measures. Individual item distributions were described in each independent random subsample using means with SD. Mardia's statistics were calculated to evaluate the multivariate behavior of the items. We verified the Kaiser–Meyer–Olkin (KMO) sampling adequacy values, the Bartlett's test of sphericity on the redundancy levels, and the matrix determinants to rule out multi-colinearity problems. To determine the underlying factorial structure of the items, we used Schwartz's Bayesian information criterion (BIC) as a dimensionality test in both subsamples.

Item–rest correlations were calculated, and an exploratory factor analysis (EFA) was performed in the first randomly selected subsample. The robust maximum likelihood (RML) method, with correction for robust mean and variance-scaled, was used for factor extraction. We tested the following models: (a) one first-order factor, as the simplest solution, taken as a reference, (b) three correlated first-order factors, maintaining the origin of the items, (c) the Schmid–Leiman solution, as an exploratory second order factor approximation, and (d) exploratory bifactor, allowing for the existence of a general common factor and three orthogonal sub-factors. Standardized factor loadings (λ), uniqueness terms (δ), latent inter-factor correlations (φ), and discrepancy values—as unstandardized residual covariance estimates were also considered. Raw loading matrices were rotated using the Promin procedure, which allows factors to be oblique to maximize factor simplicity, without assuming that all the variables are pure measures of a single dimension (57). We evaluated factorial simplicity by means of (a) the index of factor simplicity (IFS), (b) the scale fit index (SFI), (c) Bentler's scale-free matrix measure, and (d) hyperplane counts. IFS and SFI values ≥0.80 are appropriate; Bentler's measure ranges from 0 (very complex structures) to 1 (very simple ones). Hyperplane counts (loadings essentially zero except for random error) were estimated through the −0.15/+0.15 interval and through the Kaiser and Cerny procedure (58). We explored closeness to unidimensionality by means of the item residual absolute loadings (MIREAL) and the explained common variance (ECV). MIREAL is a measure of departure from unidimensionality, with values <0.30 indicating no substantial bias if a unidimensional solution is adopted (59, 60). ECV represents the proportion of common variance attributable to the general factor, which should fall within the 0.70–0.85 range if a solution is to be accepted as unidimensional (61). The effectiveness of factor scores was assessed by using the factor determinacy index (FDI). FDI is the correlation between the factor score estimates and the levels on the latent factors they estimate (62), and values of around 80 are considered adequate (63). Construct replicability is the proportion of the factor variance that can be accounted for by its indicators, which was measured by the H index (bounded between 0 and 1) with values considered appropriate when ≥0.80 (61).

A confirmatory factor analysis (CFA) using the maximum likelihood (ML) method on the second randomly selected subsample was conducted to cross-validate the factor structures obtained in the EFA.The goodness-of-fit was assessed by chi-square (χ^2^), chi-square/degrees of freedom (χ^2^/df), the comparative fit index (CFI), the Tucker–Lewis index (TLI), the root mean square error of approximation (RMSEA), and the standardized root mean square residual (SRMR). Chi-square is highly sensitive to sample size, so χ^2^/df was used, which indicates a good fit when <5 and excellent fit <3 (64, 65). CFI analyzes the model fit by examining the discrepancy between the data and the hypothesized model adjusted for the sample size, and TLI analyzes the discrepancy between the χ^2^ value of the hypothesized model and the χ^2^ value of the null model, penalizing for the number of parameters. Both CFI and TLI incremental measures indicate adequate fit when the value is >0.90, and an excellent fit when >0.95 (64, 66). RMSEA is a measurement of the error of approximation to the population, and SRMR is the standardized difference between the observed and the predicted covariance. Both RMSEA and SRMR absolute measures indicate adequate fit with values <0.08 and excellent fit when <0.06 (64, 66).

The omega composite reliability for the total scale (ω), as well as for each subscale (ω_S_), were calculated, which may be interpreted as the square of the correlation between the scale (ω)—or subscale (ω_S_)—score, and the latent variable common to the corresponding indicators (67). We also estimated the omega hierarchical (ω_H_), as the proportion of reliable variance in total scores that can be attributed to the single general factor, as well as the omega hierarchical subscale (ωHS), as the proportion of reliable variance associated with each factor (subscale) after the variance associated with the single general factor has been partitioned out (68). The average variance extracted (AVE) was also estimated, defined as the amount of variance captured by the construct vs. variance due to measurement error. Some authors suggest that the construct has convergent validity if AVE ≥ 0.50, but values of ~0.40 with a composite reliability > 0.60 are considered acceptable (69). Discriminant validity between factors exists when the AVE values are greater than the squared correlation between factors (69). The percentage of uncontaminated correlations (PUC) was estimated as the number of correlations between items from different factors divided by the total number of correlations, which indicates the proportion of correlations reflecting the possible general factor under study. When ECV and PUC values are >0.70, common variance can be regarded as essentially unidimensional, but this is also true if the PUC is <0.80 but the ECV is >0.60 and the ωH is >0.70 (61).

Finally, to assess the nomological validity of the proposed general factor, we evaluated the possible links between the general factor and the constructs of resilience and depression. To do so, we constructed a structural equation model (SEM) using the ML method. We calculated the raw inter-factor standardized latent correlations between the general factor and the other constructs, as well as the adjusted standardized links between the latent variables involved in the model. The explained variance of the latent variables (*R*^2^), as well as the unstandardized residual covariance, was estimated.

We conducted analyses for participants with non-missing data. The tests used were bilateral, and the significance level was α <0.05. The Factor (v.10.9.02) and AMOS (v.20) software packages for Windows were used to perform the statistical analysis.

## Results

### Sociodemographic Data

Table 1 shows the sociodemographic characteristics of the study participants. No significant differences were observed between the two randomly selected subsamples in terms of age, gender, educational level, or meditation experience.

### Exploratory Factor Analyses

Descriptive statistics for all items (subsample 1) are shown in Table 2. The item-rest correlations were in the same direction for all items, with values ranging from 0.42 (item NA5) to 0.67 (item NJ3). The results of the BIC dimensionality test suggested a one-factor solution (Table 3), which explained 39.3% of the total variance, with loadings (Table 2) between 0.41 (item NA5) and 0.74 (items NJ2 and NJ5). The ECV was 0.80, and the MIREAL was 0.29; thus, the unidimensionality of data was supported in the first subsample. Factor determinacy (FDI = 0.97), omega reliability (ω = 0.93), and construct replicability (*H* = 0.94) were all very good, but the goodness of model-data fit for the one-factor solution was not adequate in terms of RMSEA and SRMR values (Table 4).

**Table 2:** Descriptive statistics of the items and exploratory factor analyses (subsample 1).

				**One factor**		**Three factors**				**Schmid–Leiman solution**					**Exploratory bifactor**				
**Source**	**Item**	**Mean**	**SD**	**λ**	**δ**	**λ_1_**	**λ_2_**	**λ_3_**	**δ**	**λ_1_**	**λ_2_**	**λ_3_**	**G**	**δ**	**λ_1_**	**λ_2_**	**λ_3_**	**G**	**δ**
DE	DE1	3.28	0.81	0.48	0.77	0.44	−0.04	0.16	0.70	0.23	−0.03	0.08	0.49	0.70	0.22	−0.09	0.01	0.52	0.68
	DE2	3.44	0.92	0.57	0.68	0.44	0.10	0.12	0.64	0.23	0.08	0.06	0.54	0.64	0.24	0.06	0.01	0.53	0.64
	DE3	3.23	0.93	0.68	0.54	0.63	0.06	0.12	0.43	0.33	0.04	0.06	0.67	0.43	0.40	0.05	−0.01	0.63	0.40
	DE4	3.62	0.78	0.62	0.61	0.71	0.02	0.02	0.47	0.38	0.01	0.01	0.63	0.47	0.35	−0.06	−0.10	0.64	0.46
	DE5	3.51	0.80	0.63	0.60	0.41	0.12	0.21	0.56	0.22	0.09	0.10	0.61	0.56	0.26	0.09	0.04	0.59	0.56
	DE6	3.69	0.79	0.45	0.80	0.77	−0.11	−0.12	0.60	0.41	−0.08	−0.06	0.48	0.60	0.48	−0.08	−0.14	0.43	0.58
	DE7	3.43	1.06	0.51	0.74	0.45	−0.01	0.15	0.67	0.24	−0.01	0.07	0.51	0.67	0.32	0.01	0.04	0.47	0.67
	DE8	3.62	1.01	0.49	0.76	0.91	−0.07	−0.25	0.49	0.49	−0.05	−0.12	0.51	0.49	0.53	−0.07	−0.23	0.46	0.47
	DE9	3.84	0.86	0.65	0.58	0.66	0.04	0.07	0.46	0.35	0.03	0.03	0.65	0.46	0.39	0.01	−0.05	0.62	0.46
NJ	NJ1	3.22	1.10	0.61	0.63	0.16	0.62	−0.10	0.57	0.08	0.47	−0.05	0.45	0.57	−0.49	0.13	−0.35	0.80	0.02
	NJ2	3.68	0.96	0.74	0.45	0.12	0.69	0.03	0.39	0.07	0.52	0.01	0.58	0.39	−0.15	0.44	−0.10	0.66	0.37
	NJ3	4.05	0.97	0.70	0.51	−0.10	0.80	0.10	0.35	−0.05	0.61	0.05	0.53	0.35	−0.15	0.65	0.01	0.52	0.34
	NJ4	3.43	1.06	0.65	0.57	0.01	0.76	−0.02	0.45	0.01	0.57	−0.01	0.48	0.45	−0.18	0.54	−0.09	0.52	0.45
	NJ5	3.88	0.97	0.74	0.45	0.04	0.83	−0.02	0.29	0.02	0.62	−0.01	0.56	0.29	−0.09	0.67	−0.07	0.55	0.28
	NJ6	4.05	0.91	0.71	0.50	−0.09	0.91	−0.01	0.27	−0.05	0.68	−0.01	0.51	0.27	−0.20	0.71	−0.07	0.52	0.25
	NJ7	3.78	1.00	0.65	0.58	0.07	0.65	0.02	0.50	0.04	0.49	0.01	0.50	0.50	0.01	0.56	−0.03	0.46	0.47
	NJ8	3.56	1.15	0.63	0.60	0.01	0.80	−0.10	0.43	0.01	0.61	−0.04	0.45	0.43	−0.25	0.53	−0.15	0.53	0.43
NA	NA1	4.52	1.42	0.65	0.58	0.02	0.14	0.60	0.51	0.01	0.11	0.28	0.64	0.51	0.11	0.14	0.28	0.62	0.51
	NA2	5.22	1.04	0.54	0.71	−0.01	−0.10	0.75	0.52	−0.01	−0.08	0.35	0.60	0.52	0.21	−0.01	0.39	0.54	0.52
	NA3	4.93	1.29	0.62	0.62	0.14	−0.02	0.61	0.49	0.07	−0.02	0.29	0.65	0.49	0.23	0.03	0.30	0.60	0.49
	NA4	4.94	1.26	0.54	0.71	−0.08	−0.03	0.75	0.54	−0.04	−0.02	0.35	0.58	0.54	0.15	0.06	0.39	0.53	0.54
	NA5	5.12	1.07	0.41	0.83	0.14	−0.18	0.55	0.68	0.07	−0.14	0.26	0.48	0.68	0.23	−0.10	0.27	0.44	0.68
	NA6	4.91	1.29	0.57	0.67	−0.12	0.01	0.80	0.49	−0.06	0.01	0.37	0.61	0.49	0.09	0.05	0.40	0.58	0.49
	NA7	4.96	1.10	0.57	0.67	−0.01	0.07	0.62	0.58	−0.01	0.05	0.29	0.58	0.58	0.16	0.12	0.31	0.52	0.58

*SD, standard deviation; Characteristics of the matrix: determinant <0.001; KMO = 0.94; Bartlett's statistic = 3,702.40 (df = 276; p < 0.001): Mardia's statistic = 37.71 (p < 0.001).*

***λ**, factorial weight; δ, uniqueness; G, general factor; DE, Decentering; NJ, Non-Judging from the Five Facets of Mindfulness Questionnaire. NA, Non-Attachment*.

**Table 3:** BIC dimensionality tests.

**Subsample 1**		**Subsample 2**	
**Factors**	**BIC**	**Factors**	**BIC**
0	13,804.41	0	15,113.58
1	8,459.02*	1	8,368.37*
2	9,053.39	2	9,196.81
3	10,303.34	3	10,778.03

*Recommended number of common factors, 1; BIC, Schwartz's Bayesian information criterion.

**Table 4:** Fit indexes for the EFA, CFA, and SEM analyses.

**Group/Model**	**χ^2^**	**df**	**χ^2^/df**	**CFI**	**TLI**	**RMSEA (90% CI)**	**SRMR**
**EFA: subsample 1**							
One-factor	1,233.71*	252	4.90	0.946	0.941	0.092 (0.086–0.098)	0.099
Three correlated factors	377.56*	207	1.82	0.985	0.980	0.053 (0.046–0.060)	0.033
Schmid-Leiman	377.56*	207	1.82	0.985	0.980	0.053 (0.046–0.060)	0.033
Exploratory bifactor	302.45*	186	1.63	0.986	0.980	0.053 (0.046–0.060)	0.030
**CFA: subsample 2**							
One-factor	1,386.54*	252	5.50	0.713	0.686	0.122 (0.116–0.128)	0.110
Three correlated factors	432.29*	249	1.74	0.954	0.949	0.049 (0.041–0.057)	0.045
One second-order factor	432.29*	249	1.74	0.954	0.949	0.049 (0.041–0.057)	0.045
Bifactor model	362.16*	228	1.59	0.966	0.959	0.044 (0.035–0.052)	0.041
**Total sample**							
Bifactor model	439.25*	228	1.93	0.971	0.965	0.039 (0.034–0.045)	0.029
Structural model (SEM)	1,509.97*	752	2.01	0.944	0.938	0.041 (0.038–0.044)	0.046

*χ^2^, minimum value of the discrepancy; df, degrees of freedom; CFI, comparative fit index; TLI, Tucker-Lewis index; RMSEA (90% CI), root mean square error of approximation (90% confidence interval); SRMR, standardized root mean square residual.*

**Figure 1:**
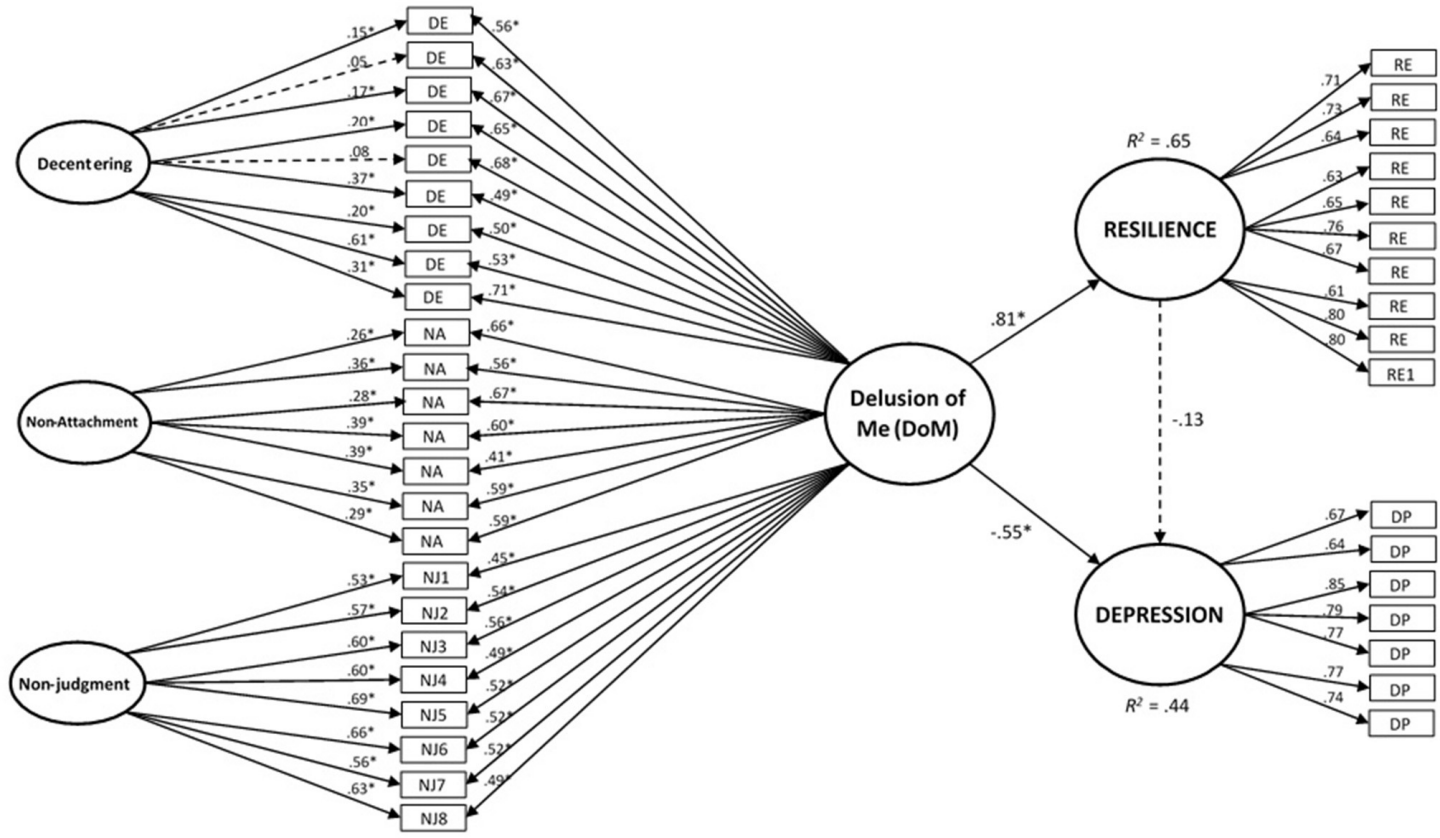
Nomological validity of the “Delusion of Me” (DoM) general factor regarding resilience and depression. Structural equations model of the nomological validity of the “Delusion of Me” (DoM) general factor regarding the constructs of “resilience” and “depression.” The circles represent latent components, and the rectangles are observable variables (items). One-way arrows represent factor loadings (in the measurement model) or standardized slopes (in the structural model). Dashed lines represent non-significant values in the corresponding factor loadings or standardized slopes (i.e., p > 0.05). *p < 0.001.

*p < 0.001; EFA, exploratory factor analyses; CFA, confirmatory factor analyses; SEM, structural equation modeling (The graphical representation of SEM is in Figure 1).

The goodness of model-data fit for a three-factor solution was better than the previous one-factor solution used as a reference (Table 4), explaining 55.8% of the total variance; moreover, loadings were congruent with the item theoretical provenance (Table 2). The uniqueness terms were lower than observed in the previous one-factor solution. The general simplicity values were adequate (see Supplementary Material). The FDI (DE = 0.94; NJ = 0.97; NA = 0.94), construct replicability (DE: H = 0.89; NJ: H = 0.93; NA: H = 0.88), and reliability (DE: ω_S_ = 0.87; NJ: ω_S_ = 0.92; NA: ω_S_ = 0.85) were appropriate. Inter-factor latent correlations were “DE–NJ” φ = 0.56, “DE–NA” φ = 0.75, “NJ–NA” φ = 0.58), and AVE was 0.50. Residual covariances were low, with average absolute values <0.001.

The Schmid–Leiman approximation presented the same explained variance, uniqueness terms, and fit to the data as the previous three-factor solution. Loadings in the general factor ranged from 0.45 (items NJ1 and NJ8) to 0.67 (item DE3), with positive weights in all of the corresponding theoretical provenance factors. The pure exploratory bifactor model explained 59.6% of the total variance and improved the fit to the data (Table 4). The uniqueness terms of the DE and NJ items were decreased, while the NA terms were exactly the same (Table 2). The general factor (G) presented loadings between 0.43 (item DE6) and 0.80 (NJ1); the AVE was 0.53. The FDI values were appropriate (DE = 0.92; NJ = 0.92; NA = 0.84; G = 0.98). The construct replicability of DE (H = 0.85), NJ (H = 0.84) and the general factor (H = 0.96) were good, but the value of the NA subscale was fair (H = 0.71). The omega hierarchical of the general factor was good (ω_H_ = 0.80), but the values of the subscales were low (DE: ω_HS_ = 0.26; NJ: ω_HS_ = 0.43; NA: ω_HS_ = 0.23). Residual covariances were very low, with average absolute values <0.001.

### Confirmatory Factor Analyses

Descriptive statistics for all items (subsample 2) are shown in Table 5. The BIC dimensionality test in subsample 2 also resulted in a one-factor solution (Table 3) that explained 41.1% of the total variance, with significant loadings in all cases (Table 5), ranging from 0.37 (item NA5) to 0.80 (item NJ3). However, the one-factor solution had a poor fit to the data (Table 4), as in the previous EFA, and thus, it was discarded.

**Table 5:** Descriptive statistics of the items and confirmatory factor analyses (subsample 2).

				**One factor**		**Three factors** ^**†**^		**Bifactor model**		
**Source**	**Item**	**Mean**	**SD**	**λ**	**δ**	**λ specific**	**δ**	**λ specific**	**G**	**δ**
EQ	DE1	3.30	0.83	0.50***	0.75	0.62***	0.62	0.18*	0.58***	0.63
	DE2	3.42	0.90	0.57***	0.68	0.66***	0.56	−0.01	0.69***	0.52
	DE3	3.18	0.88	0.50***	0.75	0.69***	0.52	0.19**	0.65***	0.55
	DE4	3.53	0.86	0.49***	0.76	0.64***	0.59	0.26***	0.58***	0.60
	DE5	3.47	0.83	0.61***	0.63	0.71***	0.50	−0.06	0.77***	0.40
	DE6	3.65	0.74	0.46***	0.79	0.54***	0.71	0.32***	0.47***	0.68
	DE7	3.37	1.05	0.39***	0.85	0.56***	0.69	0.20**	0.52***	0.69
	DE8	3.51	0.96	0.54***	0.71	0.68***	0.54	0.51***	0.57***	0.42
	DE9	3.78	0.77	0.60***	0.64	0.79***	0.38	0.45***	0.70***	0.31
NJ	NJ1	3.21	1.07	0.67***	0.55	0.73***	0.47	0.62***	0.40***	0.37
	NJ2	3.68	1.01	0.75***	0.44	0.80***	0.36	0.63***	0.48***	0.31
	NJ3	3.97	1.03	0.80***	0.36	0.83***	0.31	0.62***	0.55***	0.37
	NJ4	3.39	1.08	0.76***	0.42	0.80***	0.36	0.62***	0.50***	0.23
	NJ5	3.80	0.99	0.79***	0.38	0.87***	0.24	0.74***	0.47***	0.30
	NJ6	3.87	0.98	0.79***	0.38	0.84***	0.29	0.66***	0.52***	0.34
	NJ7	3.77	1.01	0.78***	0.39	0.81***	0.34	0.62***	0.52***	0.29
	NJ8	3.54	1.11	0.79***	0.38	0.84***	0.29	0.66***	0.53***	0.46
NAS	NA1	4.58	1.35	0.56***	0.69	0.75***	0.44	0.29***	0.68***	0.45
	NA2	5.13	1.12	0.52***	0.73	0.65***	0.58	0.30***	0.57***	0.59
	NA3	4.94	1.25	0.53***	0.72	0.74***	0.45	0.32***	0.66***	0.46
	NA4	4.80	1.25	0.61***	0.63	0.73***	0.47	0.37***	0.62***	0.48
	NA5	4.96	1.10	0.37***	0.86	0.51***	0.74	0.47***	0.35***	0.65
	NA6	4.81	1.17	0.52***	0.73	0.66***	0.56	0.43***	0.53***	0.53
	NA7	4.82	1.08	0.54***	0.71	0.67***	0.55	0.31***	0.59***	0.56

*SD, standard deviation; Characteristics of the matrix, determinant <0.001; KMO = 0.95; Bartlett's statistic = 4,108.80 (df = 276; p < 0.001), Mardia's statistic = 31.98 (p < 0.001); λ, loading; δ, uniqueness; G, general factor; EQ, experiences questionnaire; NJ, non-judging (from the FFMQ); NAS, non-attachment scale.*

The one second-order model showed the same specific loadings and uniqueness terms as the three-factor model, *p < 0.001, **p < 0.005, *** p < 0.05.

The three-correlated factor solution explained 58.4% of total variance. It showed adequate loadings (Table 5; all significant: *p* < 0.001), ranging from 0.51 (item NA5) to 0.87 (item NJ5). The fit to the data was acceptable (Table 4). The FDI values were good (DE = 0.94; NJ = 0.97; NA = 0.93). Construct replicability and reliability estimates were appropriate (DE: H = 0.88, ω_S_ = 0.87; NJ: H = 0.94, ω_S_ = 0.94; NA: H = 0.96, ω_S_ = 0.85). Inter-factor latent correlations were as follows: “DE–NJ” φ = 0.58, “DE–NA” φ = 0.80, “NJ–NA” φ = 0.57; the AVE was 0.52. Residual co-variances were low and equally distributed among all the items, with an average absolute value of 0.04.

The single second-order factor solution presented the same fit, factor loadings, and uniqueness terms as that obtained in the three-correlated factor solution, with high and significant second-order loadings [DE (γ = 0.90, *R*^2^ = 0.81, *p* < 0.001), NJ (γ = 0.64, *R*^2^ = 0.41, *p* < 0.001), NA (γ = 0.89, *R*^2^ = 0.79, *p* < 0.001)], and a total omega value of ω = 0.95. The bifactor model improved the fit to the data (Table 4), and the general factor (G) showed positive and significant loadings in all the items (Table 5), ranging from 0.35 (item NA5) to 0.77 (DE5). The averaged variance extracted (AVE = 0.54), construct replicability (H = 0.93), factor determinacy (FDI = 0.89), explained common variance (ECV = 0.61), and omega hierarchical (ω_H_ = 0.79) of the general factor were appropriate, with a PUC of 0.69. By contrast, the partialized subscales were weak in terms of construct replicability and factor determinacy, except for NJ (DE: H = 0.48, FDI = 0.32; NJ: H = 0.86, FDI = 0.77; NA: H = 0.52, FDI = 0.49). The omega hierarchical values of the subscales were also insufficient (DE: ω_HS_ = 0.11; NJ: ω_HS_ = 0.59; NA: ω_HS_ = 0.24). Residual covariances were low and equally distributed among all the items, with an average absolute value of 0.02.

### Nomological Validity

Given the appropriateness of the general factor (G) resulting from the bifactor model, we tested the nomological validity of this factor for resilience and depression in the full sample (*n* = 608). As shown in Table 4, the measurement bifactor model of the new construct fit the data well in the total sample. In addition, the fit indexes of the structural nomological model were acceptable, with the following values: χ^2^ = 1,509.97; *df* = 752 (*p* < 0.001); χ^2^/*df* = 2.01; CFI = 0.944; TLI = 0.938; RMSEA (90% CI) = 0.041 (0.038–0.044); and SRMR = 0.046. The raw inter-factor latent correlations between the constructs showed the following values: “G–resilience” φ = 0.81, *p* < 0.001; “G–depression” φ = −0.66, *p* < 0.001; “resilience–depression” φ = −0.58, *p* < 0.001). The results of the structural model are represented in Figure 1. As that figure shows, all the factorial loadings of the general factor (G) resulting from the bifactor model, as well as the factorial loadings of the other constructs (e.g., resilience and depression), were significant. The explanatory power of the general factor (G) on resilience and depression were, respectively, *R*^2^ = 0.65 (β = 0.81, *p* < 0.001) and *R*^2^ = 0.44 (β = −0.55, *p* < 0.001). The adjusted link between resilience–depression showed a non-significant value (β = −0.13, *p* = 0.070). Residual co-variances were low and equally distributed among all the items, with a mean absolute value of 0.03.

## Discussion

The main objective of this study was to test the hypothesis that there is an underlying general factor beyond the constructs of acceptance, decentering, and non-attachment. Despite the apparent semantic differences among them, they have shown to be psychometrically closely related (31, 70). Our findings suggest that a more general factor that subsumes all three of these components appears to exist. Considering the content of the referred domains and the theoretical connection among them, suggested by contemplative authors (71–73), we decided to label this general factor “Delusion of Me” (DoM) (Figure 1).

This idea of a general factor was explored and confirmed through various analytical models applied to two distinct, randomly selected subsamples. Considering the three correlated, first-order factor model and following the criteria proposed by Fornell and Larcker (69), we established convergent validity among the original components (non-judgment, decentering, and non-attachment). However, discriminant validity was only observed between decentering and non-judgment, and between non-attachment and non-judgment; in other words, decentering and non-attachment appear to be very closely related facets. The bifactor approximation was the model that best fit the data. Based on this model, the ECV—given the context of the PUC and ω_H_ values obtained—suggested the one-dimensional nature of the pool of items, with adequate loadings on the general factor and appropriate fit indexes (61). Simplicity and factor determinacy were good, and the factor score estimates unambiguously reflected the latent levels of the general factor they attempted to estimate (62). The AVE, ω_H_, and the H index were also appropriate, indicating that the general factor was well-defined (61). However, the original theoretical subscales (e.g., non-judgment, decentering, and non-attachment) remained marginal in practically all psychometric indexes after adjusting for the general factor. This suggests that the unique characteristics of each may be minimal when compared with the shared common aspects present in the general factor that seems to group them.

We have seen that several statistical indices support the unidimensional construct represented by the item pools of the self-report measures of non-judging, decentering, and non-attachment. However, failure to obtain an excellent fit for a single factor model and equally excellent fit for three correlated factors and a second-order factor (linked with the first-order factors of non-judging, decentering, and non-attachment) suggests plausible alternative hypotheses. For instance, the best fit of the bifactor model does not deny the observation of an excellent fit for the three-factor structure with a second-order factor of DoM (that can be obtained as an aggregate of the scores of the three factors). Furthermore, in the bifactor model, items of all the three factors loaded significantly on the respective latent factors (except two items of decentering) even after fragmenting the general factor of the DoM. Therefore, this also may be considered to support the notion that the constructs of non-judging, decentering, and non-attachment, even though correlating with each other and jointly representing the higher order constructs of the DoM, also represent constructs closely related to mindfulness itself.

The term “delusion” is related to the lack of decentering. Cognitive fusion refers to a mental process in which verbal events (i.e., thoughts) prevail over direct experience in terms of influence on behavior (46). As a result, the individual begins to respond to mental constructs as if they were physical facts (25, 74). On the contrary, decentering enables the individual to let go of needless entanglement with private experiences (46) such are thoughts and emotions. Decentering includes three separate components: meta-awareness of subjective experience, reduced reactivity to thought content, and dis-identification from internal experience (75). Decentering allows us to step back from an experience to examine it as separate from the self and to facilitate dis-identification from internal experience. In Buddhism and contextual sciences this conceptual self is an illusion (46, 73, 76, 77). People describe themselves in terms of their roles, history, attributes, and dispositions that blended all together constitute our narrative self that, through fusion, became “facts” (46). Although illusory, this entity has intentionality, an interest in obtaining certain results and not others (78). This fundamental mismatch between this simplified way of perceiving the world, based on personal preferences, and the true nature of reality is what Buddhism calls “ignorance” (72). Ignorance refers to the illusion that—beyond our preferences, impressions, or mental events—there is something that constitutes a separate entity, which could be called a self (78). This “mistakenly reified self-image” (44) emerges out of the “mental simulation” conforming the self.

The closest related psychological term to DoM is the self-as-a-content, a construct pertaining to Acceptance and Commitment Therapy (46), in which any private experience or content becomes the self by fusion or identification. In other words, our self becomes our current particular content of mind. When I say “my body,” the self becomes the owner of the body. The self is our vision when “I see…” and my thought when I say, “I think…” (79). When an individual identifies with a particular self-concept (i.e., I'm the kind of person who …) alternative views are less likely to be seen and inconsistencies are threatening (39, 46).

Interestingly, narrative self that could be considered a by-product of self-as-content, has been linked to medial prefrontal cortex (MPFC) activity while the experiential self, momentary self-reference centered on the present without selecting any sensory object, with pervasive reductions in the same area and increased engagement of a right lateralized network (44). MPFC, among other areas, and the left hemisphere dominance on “autobiographical-self” vs. non-verbal bodily processes or “core self” was also pointed out in later research, also targeting sources of self-reference (80). On the other hand, decentering from default mode of egocentricity was associated with greater response within the posterior insula, supramarginal gyrus, and ventrolateral prefrontal cortex, with a tendency toward greater right hemisphere involvement (81).

The essence of attachment relies on grasping or clinging to things, others, or even our own self (73). Sahdra et al. (29, 31) stated that attachment requires being stuck or fixated on ideas, images, or sensory objects, and feeling an internal pressure to acquire, hold, avoid, or change them. By contrast, as previously stated, acceptance could be defined as “experiencing events fully and without defense, as they are” (82). This also implies that the individual does not try to change, avoid, or escape from a given experience (74, 83). Along these lines, the idea of grasping, clinging, or “feeling an internal pressure to” is similar to the absence of acceptance and similar to the notion of working either against or in favor of a particular outcome. Both non-acceptance and attachment have in common the transition from preference to need through fusion.

Most people are attached to their constructs of self. This is the origin of a myriad of problems (84). If their self-narrative happens to be negative, it feeds aversion and avoidance. If the self-narrative is positive, attachment arises, and people may overestimate themselves and inadvertently cause harm to themselves and others.

The DoM index enables us to measure the degree to which expectancy of a certain outcome is confounded with actual facts. More precisely, the degree to which preferences are converted into desires of influence and become strong demands to “the reality,” in other words, the overestimation of one's own capacity to alter the events.

In this sense, DoM index is also inversely related to the concept of wei-wu-wei, the Taoist notion whereby an action is performed without an intention, and the mental process is not a means to achieve any end (53), or the concept of “reflexive awareness” from Buddhism where the phenomenal form of subjectivity is presented in an intransitive way (85). The DoM index is also coherent with the idea of a “false sense of authorship” in the Vedanta tradition in which the belief that we are in control of our will is a delusion (85–87). Finally, from the contextual behavioral science perspective also, there is no agency, and every thought, as any other behavior, is defined by a limited number of variables (88). For instance, the self in Skinner's approach has to do with responding under the control of the environment (77).The data obtained in the present study indicate that DoM might have a powerful influence on suffering (considering depression as an index), even when compared with resilience. Nevertheless, additional research is needed to further explore the DoM role on psychopathology.

The DoM approach offers two significant advantages over other approaches: first, as its referents, its potential as a transdiagnostic construct. We performed a preliminary test to determine the association between the DoM index and a common clinical index (e.g., depressive symptoms). As expected, self-reported symptoms of depression were inversely and significantly related to DoM. This finding is congruent with previous studies that have consistently reported inverse correlations between acceptance, decentering, and non-attachment with depression indexes in both clinical and non-clinical populations (1, 2, 28, 29, 89). Nevertheless, future research testing the relation between the DoM and the existent psychopathology related to depression is needed to draw the potential transdiagnostic nature and utility of the construct.

As we hypothesized, there was also a strong positive and significant association between the DoM index and resilience. Resilience is a broad construct (with no single definition) that includes various aspects of psychological resistance (90). Resilience could be described as a multidimensional factor that moderates negative emotions and distress, thereby facilitating a flexible adaptation to suboptimal conditions (91). There is substantial empirical evidence that psychological resilience can help an individual to regain or maintain physical and mental health (92–94). Interestingly, when resilience, depression, and DoM were considered together (Figure 1), resilience lost its explanatory power over depression symptom scores and was submerged by the power of DoM to explain depressive mood. It should be noted that all three DoM constituents have been considered relevant in “third wave” clinical approaches. For instance, in Acceptance and Commitment Therapy, flexible and healthy psychological functioning relies on the six corners of the hexaflex model (i.e., flexible attention to the present moment, acceptance, defusion, self as a context, values, and committed action) two of which are contained in DoM (46). Along with ACT, therapies such MBCT, mindfulness-based relapse prevention (MBRP) and DBT exactly or similar terms such as non-reactivity, non-judgmental, aversion, craving, acceptance, willingness, “letting go,” decentering, or defusion are defined and specifically targeted within the therapy process. Congruently, the three DoM constituents have been studied as mechanisms of change for “third wave” clinical approaches when targeting a wide diversity of mental disorders such as depression, anxiety, substance use disorders, and personality disorders (95–98).

In this regard, our findings suggest that, by training one of these facets, the individual would actually train all three. If this is true, it could help to tailor the individual's treatment according to their specific needs and preferences. For example, a given individual may be reluctant to practice mindfulness of thoughts to promote decentering, but could be more open to psycho-education to foster acceptance, or to self-distancing training (89) and track the impermanence of experiences (90) to increase defusion or to random acts of kindness (99) to encourage non-attachment. In this regard, future studies should analyze the utility and the potential therapeutic value of this new construct. As Fahlberg and Fahlberg (100) suggest, if we could only weaken the hold of our conditioned thoughts, emotions, and behaviors this could lead to a more pro-social and rational life and therefore mitigate the negative social and environmental consequences of egoic behavior and lifestyles (101). The second strength of the DoM index is that it appears to be psychometrically tenable. As we stated in the introduction, the currently available measures of mindfulness are inconsistent and poorly correlated. In a recent review, Park et al. (14) concluded that “…the current mindfulness scales have important conceptual differences and none can be clearly recommended based on their psychometric properties. Researchers should proceed with caution before optimizing any mindfulness intervention based on existing scales.” In this regard, approximately half of the studies involving mindfulness-based interventions either fail to show any significant pre-post treatment effect in self-reported measures of mindfulness, or the outcomes are no better than those obtained with other active interventions (102), pointing to weaknesses in the tools used to measure the effects of mindfulness practice. We consider DoM as a trait measure, since EQ, NAS, and FFMQ fail on this category. Similarly, we also think on DoM, mainly, as a by-product of mindfulness practice since Decentering and Non-attachment can be considered as consequences of mindfulness (27, 70). On the other hand, from a Western point of view of mindfulness, acceptance is part of it, rather than an aftermath (7). Although our study does not include any pre-post assessment, we think that it is highly likely that the DoM index will be sensitive to change given the strong sensitivity of its components (i.e., EQ, NAS, and Non-Judgment from FFMQ) (26, 27, 103).

DoM intends to be useful in assessing interventions targeting “ego.” The identification with thoughts that represent things and ideas transfer the sense of identity from them to our self. As Tolle (104) pointed out, when “my toy” is broken or taken away, intense suffering arises, not because of the intrinsic value of the toy, but because of the thought of “mine.” The toy becomes part of the child's developing sense of self, of “I,” and, as we see, the pronoun “mine” is inseparable of the attachment quality. Finally, attachment and lack of acceptance seem to be the heads and tails of the same phenomenon, and have the outcomes that “I” want.

Recently, it has been proposed the term “hypo-egoic” to describe states where (1) conceptual self-awareness is low; (2) the phenomenal self is not highly individuated from its context; and (3) the person is not selfishly invested in the outcome of a particular situation (105). The usual egoic form of self contributes to depression, anxiety, anger, jealousy, and other negative emotions that we experience. It enables us to ruminate and, interpersonally, undermine our relationships, and it can be a source of social conflict by promoting dislike for those who are different from us (106).

In summary, DoM index is psychometrically robust and could be a reliable measure in mindfulness field. The construct may function as a transdiagnostic index to measure the overestimation of one's own capacity to alter the events. DoM appears to be powerful connected to both negative affect and resilience. Additionally, and beyond the strictly individual assessment, the construct has the potential to be useful in relation to prosocial and lifestyle variables and in the context of interventions designed to induce a “hypo-egoic” effect.

## Limitations and Future Directions

The main limitation of the present study is that the results are based only on a limited number of instruments, despite the wide variety of scales that could have been used to assess these three constructs. Moreover, other constructs such as equanimity or compassion could have been included in the model, as these have indirect implications for self-concept and self-reference (79). In addition, all of the measures used in this study were self-reported, with the inherent potential for bias such as social desirability or acquiescence. In particular, the possibility of “common method bias” should not be discarded, as using the same methodology to assess different constructs often loads on a single factor due to the similarity of the assessment, and not because of content overlap between the constructs. The participant recruitment and methodology of survey over regional online websites and social media could have also hamper the generalizability of our results. Another limitation is that, although we assessed the association between DoM and depression, our sample did not include clinical diagnosis, and therefore, we do not know the clinical applicability of our findings. In addition, the study design does not allow us to make causal inferences between variables, and also the role of possible confounders was not evaluated. Nevertheless, it is worth highlighting that one important strength was the use of two randomly selected sub-samples to cross-validate results obtained.

Future research must consider other outcomes related to this construct, ideally including ecological measurement models of mindfulness in daily life. Additionally, the utility of the DoM concept in clinical samples should be tested in future research, especially through longitudinal studies to test causal inferences. In summary, DoM emerges as a robust, potentially transdiagnostic concept, with possible influences on well-being, but further studies should analyze its utility at the therapeutic level.

## Conclusions

The study shows that there is a general factor that subsumes the constructs of acceptance, decentering, and non-attachment. We used analytical models applied to two distinct, randomly selected subsamples, but despite the apparent semantic differences among them, they have shown to be psychometrically closely related. Thus, we have labeled it as “Delusion of Me” (DoM) to refer to the delusion of control of our will, of our “self.” This construct appears to correlate negatively to psychopathology and positively to resilience.

## Data Availability Statement

The original contributions presented in the study are included in the article/Supplementary Material, further inquiries can be directed to the corresponding authors.

## Ethics Statement

The study protocol was approved by the corresponding regional health authority, the Aragon Ethics Committee (CEICA), Spain (registry: PI12/00083) and all participants signed a consent form indicating their willingness to participate. Participants were informed about the purpose of the study and informed that all response would be treated confidentially.

## Author Contributions

JS, AC, and JG-C designed the study. ED-C and SG executed the fieldwork. JM-M performed the data analyses. JS and JM-M were in charge of writing the manuscript and responding to reviewers comments. JG-C, AC, and BA assisted in the editing of the manuscript. All authors read and approved the final version of the manuscript.

## Funding

JM-M was supported by the Wellcome Trust Grant (104908/Z/14/Z). Additionally, Ciberobn is an initiate of the ISCIII.

## Conflict of Interest

The authors declare that the research was conducted in the absence of any commercial or financial relationships that could be construed as a potential conflict of interest.

## Publisher's Note

All claims expressed in this article are solely those of the authors and do not necessarily represent those of their affiliated organizations, or those of the publisher, the editors and the reviewers. Any product that may be evaluated in this article, or claim that may be made by its manufacturer, is not guaranteed or endorsed by the publisher.
