# Supplementary material for: Decentering, Acceptance, and Non-Attachment: Challenging the Question “Is It Me?”
Source: Front Psychiatry. 2021 Nov 18;12:659835. doi: 10.3389/fpsyt.2021.659835 (PMC8637104; doi:10.3389/fpsyt.2021.659835)
Supplement: Supplementary file 1 [file Data_Sheet_1.DOCX]

The selection of 24 items which are the components of the DoM construct

Items coming from NAS-7

| **1** | (1) | Puedo dejar ir los sentimientos de resentimiento e insatisfacción sobre el pasado. |
| --- | --- | --- |
| **2** | (2) | Puedo disfrutar de experiencias agradables sin necesitar que duren siempre. |
| **3** | (3) | Considero los problemas que me afectan como cosas en las que trabajar,  en lugar de como razones para desmoralizarme o desmoronarme. |
| **4** | (4) | Puedo disfrutar de mi familia y amigos sin sentir la necesidad de aferrarme a ellos. |
| **5** | (5) | Puedo alegrarme de los éxitos de los demás sin sentir envidia. |
| **6** | (6) | No me quedo enganchado en desear una vida “ideal” o “perfecta”. |
| **7** | (7) | Cuando una buena experiencia termina me quedo bien pasando a lo que venga luego. |

Items coming from EQ

| **1** | (1) | Soy más capaz de aceptarme a mí mismo como soy. |
| --- | --- | --- |
| **2** | (3) | Me doy cuenta de que no me tomo las dificultades de forma tan personal. |
| **3** | (4) | Puedo separar mis pensamientos y sentimientos de mi mismo. |
| **4** | (5) | Puedo tomarme tiempo para responder a las dificultades. |
| **5** | (7) | Puedo observar sentimientos desagradables sin ser arrastrado hacia ellos. |
| **6** | (8) | Tengo la sensación de que soy completamente consciente de lo que está sucediendo a mi alrededor y dentro de mí. |
| **7** | (9) | Veo que, en realidad, no soy mis pensamientos. |
| **8** | (10) | Soy consciente de sentir mi cuerpo como un todo. |
| **9** | (11) | Veo las cosas desde una perspectiva más amplia. |

Items coming from Non-Judge from FFMQ

| **1** | (3) | Me critico a mi mismo/a por tener emociones irracionales o inapropiadas. |
| --- | --- | --- |
| **2** | (10) | Me digo a mi mismo/a que no debería sentir lo que siento. |
| **3** | (14) | Creo que algunos de mis pensamientos no son normales o son malos y que no debería pensar así. |
| **4** | (17) | Hago juicios sobre si mis pensamientos son buenos o malos. |
| **5** | (25) | Me digo a mi mismo/a que no debería pensar como pienso. |
| **6** | (30) | Creo que algunas de mis emociones son malas o inapropiadas y que no debería sentirlas. |
| **7** | (35) | Cuando tengo pensamientos o imágenes perturbadoras, me juzgo como bueno o malo, dependiendo del contenido. |
| **8** | (35) | Me critico cuando tengo ideas irracionales. |
|  |  |  |

*Numbers in bold refer to the number of the item used in the present study, while numbers in brackets refer to the number from the original scales they were taken from.
