# Supplementary material for: Decentering, Acceptance, and Non-Attachment: Challenging the Question “Is It Me?”
Source: Front Psychiatry. 2021 Nov 18;12:659835. doi: 10.3389/fpsyt.2021.659835 (PMC8637104; doi:10.3389/fpsyt.2021.659835)
Supplement: Supplementary file 2 [file Data_Sheet_2.docx]

**SUPPLEMENT**

*General simplicity values (Exploratory Factor Analyses)*

The general simplicity values for a three-factor solution were adequate (IFS = .95; SFI = .91; Bentler = .99), with loadings in the hyperplane (-.15 / +.15) = 43 (59.7%): the Kaiser-Cerny hyperplane count (cut-offs: DE = .20; NJ = .21; NA = .19) was 46 (63.9%), and the ideal hyperplane count was 48 (66.7%).
